# Sequencing and G-Quadruplex Folding of the Canine Proto-Oncogene *KIT* Promoter Region: Might Dog Be Used as a Model for Human Disease?

**DOI:** 10.1371/journal.pone.0103876

**Published:** 2014-08-01

**Authors:** Silvia Da Ros, Eleonora Zorzan, Mery Giantin, Lara Zorro Shahidian, Manlio Palumbo, Mauro Dacasto, Claudia Sissi

**Affiliations:** 1 Dept. of Pharmaceutical and Pharmacological Sciences, University of Padova, Padova (PD), Italy; 2 Dept. of Comparative Biomedicine and Food Science, University of Padova, Legnaro (PD), Italy; Imperial College London, United Kingdom

## Abstract

Downregulation of gene expression by induction of non-canonical DNA structures at promotorial level is a novel attractive anticancer strategy. In human, two guanine-rich sequences (h_kit1 and h_kit2) were identified in the promotorial region of oncogene *KIT*. Their stabilization into G-quadruplex structures can find applications in the treatment of leukemias, mastocytosis, gastrointestinal stromal tumor, and lung carcinomas which are often associated to c-kit mis-regulation. Also the most common skin cancer in domestic dog, mast cell tumor, is linked to a mutation and/or to an over-expression of c-kit, thus supporting dog as an excellent animal model. In order to assess if the G-quadruplex mediated mechanism of regulation of c-kit expression is conserved among the two species, herein we cloned and sequenced the canine *KIT* promoter region and we compared it with the human one in terms of sequence and conformational equilibria in physiologically relevant conditions. Our results evidenced a general conserved promotorial sequence between the two species. As experimentally confirmed, this grants that the conformational features of the canine kit1 sequence are substantially shared with the human one. Conversely, two isoforms of the kit2 sequences were identified in the analyzed dog population. In comparison with the human counterpart, both of them showed an altered distribution among several folded conformations.

## Introduction

DNA double helix structure is based on Watson-Crick base-pairing. Nevertheless, different base pairing can form and lead to several other “non canonical” structures. A relevant example is provided by guanines which can pair by means of eight hydrogen bonds between the N1 and N2 on Watson-Crick face and the O6 and N7 on Hoogsten face, thus forming a planar G-tetrad [1]. Consecutive G-tetrads can stack one over each other providing very stable supramolecular structures called G-quadruplexes (G-4). This architecture is further stabilized by coordination of cations, such as K^+^ and Na^+^, that neutralize the negative charge density deriving from the guanines O6 carbonyl groups pointing towards the central core of each tetrad.

Accordingly, to potentially fold into a stable G-4 structure a nucleic acid sequence must be G-rich. In cells, sequences fulfilling this requirement are represented by the telomeres, noncoding regions located at the chromosomes end that, in vertebrates, are constituted by d(TTAGGG) repeats. Additionally, bioinformatics analyses on the human genome identified a relevant number of putative G-4 forming sequences clustered at defined regions such as the transcription start site, the 5′-UTR, and the 5′ end of the first intron, whereas they are depleted in coding regions [2], [3]. Interestingly, they have also been identified upstream of gene transcription start sites of oncogenes such as *c-MYC*, *KRAS*, *BCL-2*, *VEGF* and *PDGF* but not at oncosuppressor genes level. This conserved localization suggested that G-4 may play functional role in the regulation of gene expression; this hypothesis is now supported by increasing evidences that G-4 actually form in living cells and are critical for genome integrity [4], [5]. Moreover, G-4 occurrence at promotorial sites prevents the correct assembly of the transcriptional machinery and leads to transcription alteration and inhibition of gene expression. For these reasons, G-4 stabilization of oncogenic promoters represents a potential therapeutic intervention to obtain antiproliferative effects [6], [7].

Recently, two G-rich sequences, human kit1 (h_kit1) and kit2 (h_kit2), were identified in the promotorial region of the human oncogene *KIT* [8]. They occur respectively between position −12 and −34 bp and between position −64 and −84 bp upstream the transcription start site (5′-UTR) [9]. Both these sequences fold into G-4 and the resulting molecular structures were solved by NMR and X-ray crystallography [10]–[13].

The *KIT* gene encodes a type III tyrosine kinase receptor, c-kit, which is the 145 kDa glycoprotein receptor of stem cell factor (SCF) [14], [15]. Binding of SCF to c-kit leads to receptor homodimerization, autophosphorylation and, consequently, to initiation of intracellular signaling pathways such as Shc/Ras/MAPK, JAK/STAT and PI3K cascades. In human, development of melanocytes, erythrocytes, germ cells, mast cells and interstitial cell of Cajal occur through SCF-kit interaction, while ligand-independent auto-phosphorylation of c-kit is typical of a number of tumors, such as leukemias, mastocytosis, gastrointestinal stromal tumor (GIST) and lung carcinomas [16]–[18].

To solve the consequences of c-kit mis-regulation, numerous tyrosine kinase inhibitors (TKIs) have been developed and are now clinically used for the treatment of several malignances. The now-a-day available pharmacological therapy is based on the chemotherapeutics imatinib (Glivec/Gleevec; Novartis) and sunitinib (Sutent; Pfizer). However, mutations either in the juxtamembrane domain (exon 11), in the extracellular domain (exon 9), in the cytoplasmatic ATP-binding domain (exon 13/14) or in the activation loop domain (exon 17) are frequently occurring and are responsible for resistance against TKIs [19], [20].

An alternative approach to overcome these resistance problems rests in driving a downregulation of *KIT* gene expression at the transcriptional level. As a proof of concept, some ligands were designed to selectively target the G-4 forms of h_kit1 or h_kit2. So far, several small molecules belonging to different scaffolds have been demonstrated to bind one G-4 structure of *KIT* and to reduce basal levels of c-kit expression in a dose-dependent manner and in different cell lines [9], [21]–[25]. On the contrary, a triarylpyridine derivative was found to reduce the stability of G-4 in *KIT* and increments *KIT* gene expression in HGC-27 cells [26]. In addition, both synthetic DNA (PNA) and peptide (peptidomimetic) analogues were found to be able to efficiently recognize h_kit1 [27], [28].

Naturally occurring cancers in pet dogs and humans share many features, including histological appearance, tumor genetics, potential prognostic and therapeutic molecular targets as well as response to conventional therapies. As a consequence, the domestic dog is nowadays considered as an excellent translational animal model for human oncology [29]–[32].

Mast cell tumor (MCT) is the most common skin cancer in dogs (7–21% of all canine cutaneous tumors), but it is likely to arise in extra-cutaneous sites including the gastrointestinal tract (visceral MCT). Although the etiology of canine MCT is still unknown, several studies revealed that this pathology is linked to a mutation and/or to an over-expression of c-kit [33], [34]; furthermore, c-kit mutations occurring in dog MCTs are similar to those found in human GIST [35]. This has made MCTs an important model disease to assess the functional consequences of c-kit abnormalities in cancer. Consistently, the use of TKIs today represents the available pharmacological treatment also for unresectable grade 2–3 canine MCTs sharing c-kit mutations [36].

With the aim to investigate if the promotorial region of the canine *KIT* oncogene may represent a possible new molecular therapeutic targets for comparative oncology studies, in this work we verified the existence of G-rich sequences in this genomic region and we assess their potential to fold into stable G-4 structures.

To reach this goal we cloned and sequenced part of the canine *KIT* proximal promoter region and we compared it with the human one in terms of sequence and conformational equilibria in physiologically relevant conditions.

The results herein collected will have a double valence. Indeed, they will help in assessing whether:

- the dog may represent a robust translational model for evaluating the functional consequences of c-kit expression modulation in human cancer;
- the application of selective G-4 binders as novel anticancer drugs for canine MCTs is feasible.

## Materials and Methods

### Ethics Statements

Blood samples and tissue biopsies were not taken for the purposes of this study. Tissue biopsies were recruited from dogs affected by MCT and undergoing surgery. Consent from the owners had been obtained for dogs undergoing MCT surgery.

Blood samples were collected from healthy random-source adult kennel dogs undergoing routine examination. Consent from the owners was not required, because there is an agreement between our Veterinary School and the kennel for the execution of routinary clinical check ups.

Both kennel dogs and dogs affected by MCT and undergoing surgery were under the care of licensed veterinarians and participation in the study did not influence decisions of care. Animal care, surgery and post-surgery were carried out in accordance with good veterinary practices. According to the Italian law (D. Lgs. n. 116/92), an Institutional Animal Care and Use Committee approval number and date of approval for the study are not requested for private practice. Only a written informed consent is needed to conduct a clinical trial.

### Canine samples and DNA extraction

Peripheral blood was collected in EDTA tubes from 28 healthy random-source adult kennel dogs undergoing routine examination. DNA was extracted by using the DNeasy Blood & Tissue Kit (Qiagen, Milan, Italy) according to manufacturer’s instructions. To investigate the possible occurrence of mutation in the putative G-4 sequence, 23 tissue biopsies were recruited from dogs affected by MCT and undergoing surgery and c-kit mutational analysis. Genomic DNA was extracted by using the Invisorb Spin DNA Extraction Kit (STRATEC Molecular, Berlin, Germany) in accordance with manufacturer’s proceedings. No distinctions were done in term of breed, age and any other tumor characteristics.

### Partial amplification and sequencing of the canine *KIT* proximal promoter

To identify canine G-4 sequences, human *KIT* G-4 sequences [8], [37] were aligned to canine *KIT* 5′-upstream region (chromosome 13) by using the bio-informatic tool Multalin (http://multalin.toulouse.inra.fr/multalin/).

Primers (Eurofins MWG Operon, Ebersberg, Germany) for KIT_1_F and KIT_1_R (see Table 1), spanning a 875 base pair fragment of canine *KIT* proximal promoter region, were designed by using the Primer3 software (http://primer3.sourceforge.net/). This fragment included 350 bp of *KIT* proximal promoter region, the entire 5′ UTR region, the first *KIT* exon and 424 bp of the first intron. A further couple of primers, to be used for a nested PCR (*KIT* nested, Table 1), were designed likewise.

**Table 1:** Primers for polymerase chain reaction amplification and sequencing of partial canine *KIT* proximal promoter region containing the two putative G-quadruplex sequences.

Primer	Primer sequence (5′-3′)	Expected product size (bp)
**KIT_1_F**	ACCTTATTGTCTGGGGAGCA	875
**KIT_1_R**	GCGCAACTTTCAACAAAAGG	
**KIT_nested_F**	GAGAGCCGGTGATATGCAG	296
**KIT_nested_R**	AGCAGGACGCAGAGAAAATC	

F = forward, R = reverse.

About fifty ng of genomic DNA were loaded in the first PCR reaction and amplified by using the TaKaRa LA Taq Hot Start polymerase (Takara Biotechnology, Otsu, Shiga, Japan). The reaction, carried out in a TPersonal thermocycler (Biometra GmbH, Goettingen, Germany), consisted of 0.5 µM of each primer, 2.5 U of DNA polymerase, 400 µM of deoxy-ribonucleotide triphosphate mix, 5% DMSO and 1X reaction buffer (final concentrations). The following PCR conditions were used: an activation step at 94°C for 30 sec (hot start), 30 cycles of 10 sec at 98°C, 30 sec at 60°C, 45 sec at 68°C, and a final extension step of 10 min at 72°C. Products obtained were checked in 1.5% agarose (Sigma-Aldrich Chemie GmBH, Munich, Germany) gel electrophoresis.

One µl of the undiluted PCR product obtained during the first PCR reaction was used for the nested PCR, carried out by using the same conditions described above. The PCR product was visualized in 1.5% agarose gel electrophoresis and sequenced by using the ABI 3730XL DNA Analyzer (Life Technologies, Foster City, CA). Sequences were analyzed with the FinchTV software (Geospiza Inc., Seattle WA).

### Cloning

The nested PCR product (296 bp) was cloned into the pCR2.1-TOPO plasmid by using the TOPO TA Cloning Kit (Life Technologies, Foster City, CA). Cloned plasmids were inserted in TOP10 One Shot *E. coli* (Life Technologies, Foster City, CA) and left growing in LB Agar (Sigma-Aldrich Chemie GmBH, Munich, Germany) supplemented with 5-bromo-4-chloro-3-indolyl-β-D-galactopyranoside (X-Gal, Sigma-Aldrich Chemie GmBH, Munich, Germany) and ampicillin (Sigma-Aldrich Chemie GmBH, Munich, Germany). White colonies were picked up and incubated overnight in LB Broth (Sigma-Aldrich Chemie GmBH, Munich, Germany). To isolate the plasmid, the QIAprep Spin Miniprep Kit (Qiagen, Milan, Italy) was used. To verify the presence of the fragment of interest, plasmids were digested with EcoRI and checked by agarose gel electrophoresis. Then, plasmids were sequenced with universal M13 forward and reverse primers. At least five clones from three independent PCR reactions and cloning procedures were verified by sequencing (Macrogen, Amsterdam, Netherlands).

### Statistical analysis

To evaluate the possible relationship between the presence of SNP in canine kit2 sequence and the tendency to develop MCT, a Fisher exact test was performed by using the GraphPad Prism 5 software (San Diego, California, USA). A value of P<0.05 was considered significant.

### CD spectroscopy

Circular dichroism spectra were recorded on a Jasco J-810 spectropolarimeter equipped with a Peltier temperature controller using 1–10 mm path-length cells in the 230–350 nm wavelength range. For each spectrum, 3 scans were acquired at a 50 nm/min scanning speed. Spectra were acquired in the absence and in the presence of variable KCl concentrations in 10 mM Tris, at pH 7.5. DNA substrates (Metabion International, Germany) were used at 4 µM final concentration and, before use, all DNA solutions were annealed in the required buffer. Observed CD signals were converted to mean residue ellipticity [θ]  = deg×cm^2^ x dmol^−1^ (Mol. Ellip.).

To calculate the dissociation constant for KCl (Kd), the fraction of bound DNA was calculated [AB]/[A]_tot_ = (S-S_o_)/(S_∞_-S_0_), where S_o_ and S_∞_ are the signal corresponding to the free and bound target, respectively. This was plotted as a function of salt concentration ([B_tot_]). Experimental data were fitted according to a single binding event process according to the following equation: [AB]/[A]_tot_ = [B_tot_] (1/(1+Kd)).

Melting and annealing curves were recorded by monitoring the variation of the dichroic signal at one constant wavelength (260 nm) in the temperature range 25–95°C with a temperature slope of 0.8°C/min. Melting temperatures were determined by the first derivative of the melting curves.

### Electrophoretic Mobility Shift Assay – EMSA

Oligonucleotides (5 µM) were 5′-labeled with ^32^P by 1 h incubation at 37°C with 1 µl dATP[γ−^32^P] (Perkin Elmer, Life Sciences) and 1 U of T4 Polynucleotide Kinase (Thermo Scientific) in a final volume of 50 µl of provided forward reaction buffer (Thermo Scientific). Then the enzyme was removed by two extraction with 100 µl of phenol-chloroform-isoamyl alcohol (25∶24∶1) mixture. After ethanol precipitation, DNA was solubilized in 10 mM Tris, 1 mM EDTA, pH 8.0.

Samples containing 0.1 µM of labeled oligonucleotides and increasing concentration of not labeled DNA were annealed over-night in 10 mM Tris, pH 8.0 in the presence of variable concentrations of KCl. The reaction products were resolved on a 20% PAGE acrylamide (acrylamide/bisacrylamide 19∶1) in 0.5X TBE containing 10 mM KCl. Electrophoretic run was performed at 250 Volt for 3 hours. At the end of the electrophoretic run the gel was exposed overnight on a storage phosphor screen (Amersham Pharmacia Biotech) and finally scanned with a Storm 840 (Amersham Pharmacia Biotech).

## Results

### Sequencing of the canine *KIT* promotorial region

**Figure 1:**
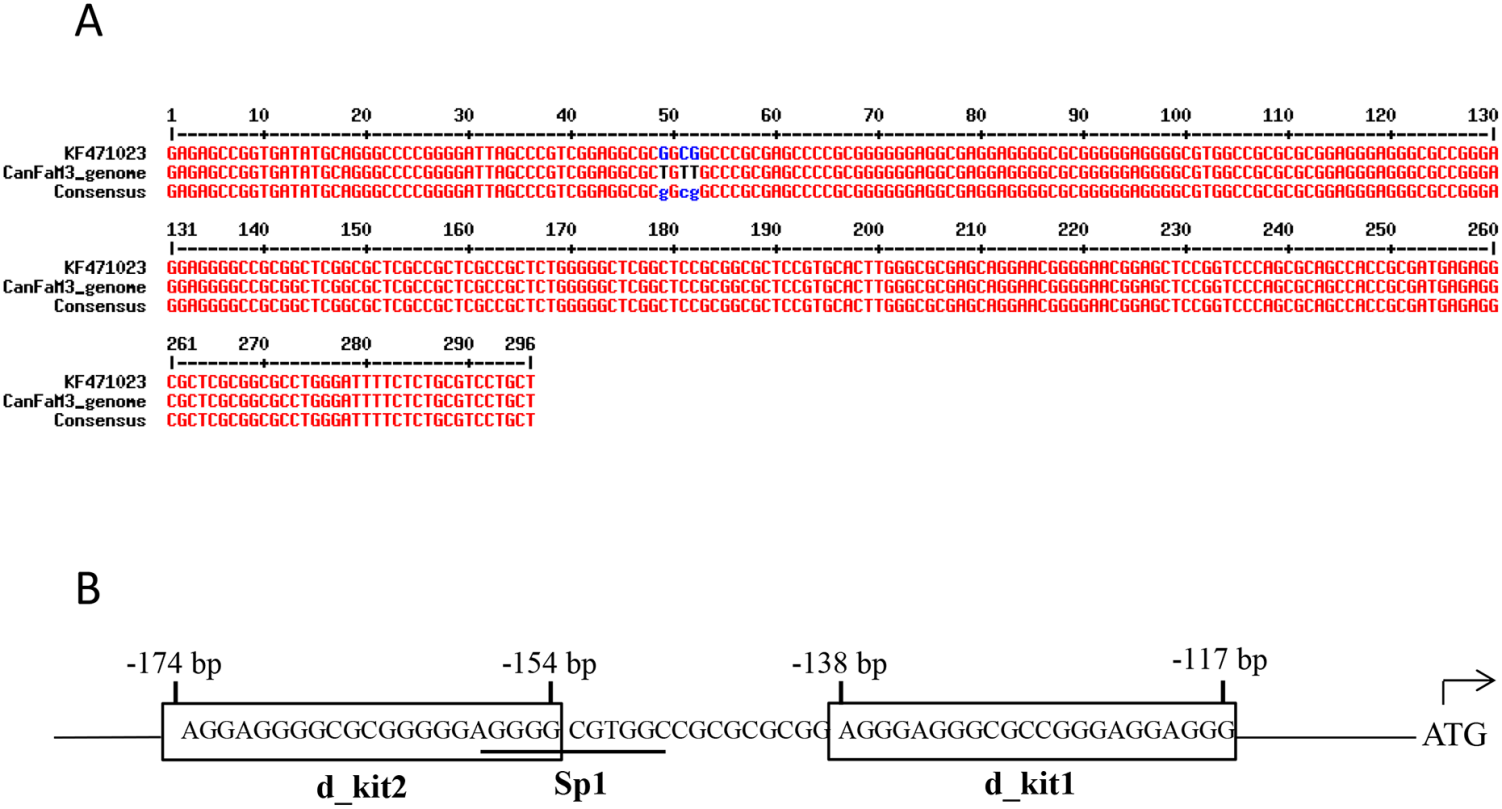
The canine *KIT* promotorial region contains two putative G-quadruplex forming regions. PANEL A: Alignment of KF471023 (canine *KIT* partial promoter sequence) and Broad CanFam 3.1/canFam3 genome (UCSC Genome Browser database). PANEL B: Canine d-kit1 and d-kit2 putative G-quadruplex folding sequences and their location within the proximal c-kit promoter, shown with respect to the first codon.

Since it is known that *KIT* may show sequence mutations in MCTs [38]–[40], samples for canine *KIT* promotorial region sequencing were collected from both healthy dogs (28 blood samples) and from animals affected by MCT (23 archival tumor biopsies). The obtained partial sequence of canine *KIT* promoter was submitted to GeneBank database (http://www.ncbi.nlm.nih.gov/) with the following accession number: KF471023. It consists in the proximal promoter region (224 bp upstream the 5′ UTR region), the entire 5′ UTR region (28 bp long) and the beginning of the first exon (until +44 bp after ATG). Overall, it differs in 3 nucleotides from Broad CanFam 3.1/canFam3 genome submitted in UCSC Genome Browser (http://genome.ucsc.edu/cgi-bin/hgBlat?command=start) (Figure 1A).

**Figure 2:**
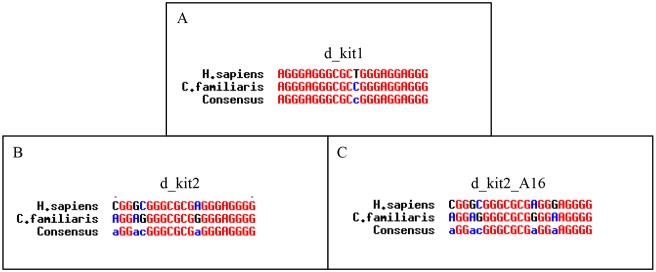
The human and canine promoter sequences show species-differences. Alignment of human G-quadruplex sequences and putative canine sequences identified by cloning and sequencing. A) d_kit1; B) d_kit2; C) d_kit2_A16.

By comparing human and canine promoter sequences, two putative portions for the G-4 folding were identified: a first one (5′-AGGGAGGGCGCCGGGAGGAGGG-3′) was attributable to h_kit1, and was located from −117 to −138 bp upstream the ATG. The second one (5′-AGGAGGGGCGCGGGGGAGGGG-3′), that might be considered as the h_kit2 counterpart, was located from −154 to −174 bp upstream the first codon (Figure 1B). Noteworthy, the positions cannot be referred to the trascription start site, because the 5′ UTR region of canine *KIT* is not defined and fully characterized in both NCBI and ENSEMBL databases (http://www.ncbi.nlm.nih.gov/, http://www.ensembl.org/index.html). Both sequences showed evident species-differences (Figure 2).

The screening of healthy and pathologic canine samples showed the presence of a single nucleotide polymorphism (SNP) in canine kit2. In particular, 32 out of 51 samples (62.7%) presented the nucleotide A in −159 position (defined from now on as d_kit2_A16), while the remaining 19 showed the nucleotide G (37.3%: named as d_kit2). The statistical analysis did not reveal a significant association between the presence of each genotype and the tendency to develop MCT (P = 0,1577, Figure 3). Since the number of tested samples was not representative of the entire population, further investigations are needed to obtain the frequency of the aforementioned SNP in the canine population.

**Figure 3:**
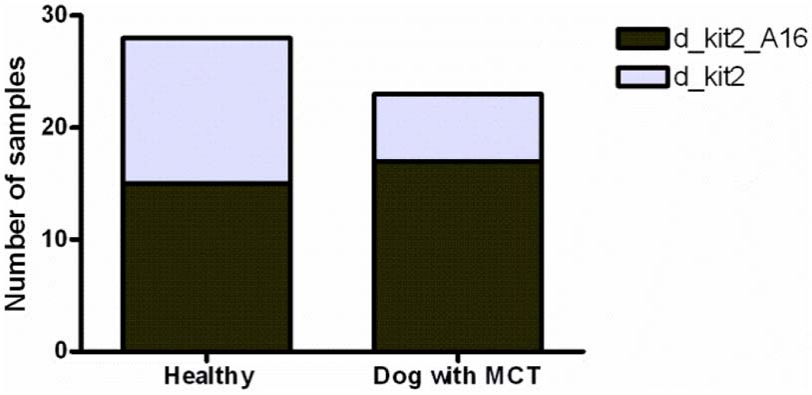
The identified (SNP) in canine kit2 is not associated to the healthy/MCT state. Association between the presence of d_kit2 or d_kit2_A16 and healthy/pathologic state in 51 canine blood and tumor samples. Fisher exact test (p = 0.1577).

Many breeds have been reported to be predisposed to MCT [41]. Breed was not considered in this study, but it could be interesting in perspective to evaluate the influence of such a genetic factor on *KIT* promoter genotyping.

### G-quadruplex formation in the canine *KIT* sequences

The alignment of the canine counterpart of the two minimal G-4 forming sequences present in the promotorial region of human *KIT* showed some point mutations between the two species (Figure 2). The structural studies available for the human sequences suggest they likely occur at different domains of the G-4 structures. In the case of canine kit1 (d_kit1) the difference involves residue 12 (T12C), which is localized in the second loop of the human parallel G-4. Thus, we do not expect remarkable modulation in the G-4 forming potential from the human to the canine sequence. Not the same occurs with the sequences related to h_kit2 where one (d_kit2) or two guanines (d_kit2_A16) involved in G-tetrad formation are converted into adenines. Thus, the conformational properties of the d-kit2 sequences might be affected by the above changes. Hence, they were investigated in deeper detail and compared to those of the human counterparts and as well as to some additional mutated sequences (Table 2).

**Table 2:** Human (h_) and canine (d_) sequences of *KIT* proximal promoter region used in this work.

**h_kit1**	AGGGAGGGCGCTGGGAGGAGGG
**d_kit1**	AGGGAGGGCGC***C***GGGAGGAGGG
**h_kit2**	CGGGCGGGCGCGAGGGAGGGG
**d_kit2**	***A*** GG***AG***GGGCGCG***G***GGGAGGGG
**d_kit2_A16**	***A*** GG***AG***GGGCGCG***G***GG***A***AGGGG
**h_kit2_T21**	CGGGCGGGCGCGAGGGAGGG***T***
**h_kit2_T12/21**	CGGGCGGGCGC***T***AGGGAGGG***T***
**d_kit2_T12/21**	***A*** GG***AG***GGGCGC***T*** **G**GGGAGGG***T***
**h_kit2_2tet**	CGG***A***CGG***A***CGCGAGG***A***AGG***AT***

Mutations with reference to the human sequences are in bold and italic.

### Canine and human kit1 share common conformational features

**Figure 4:**
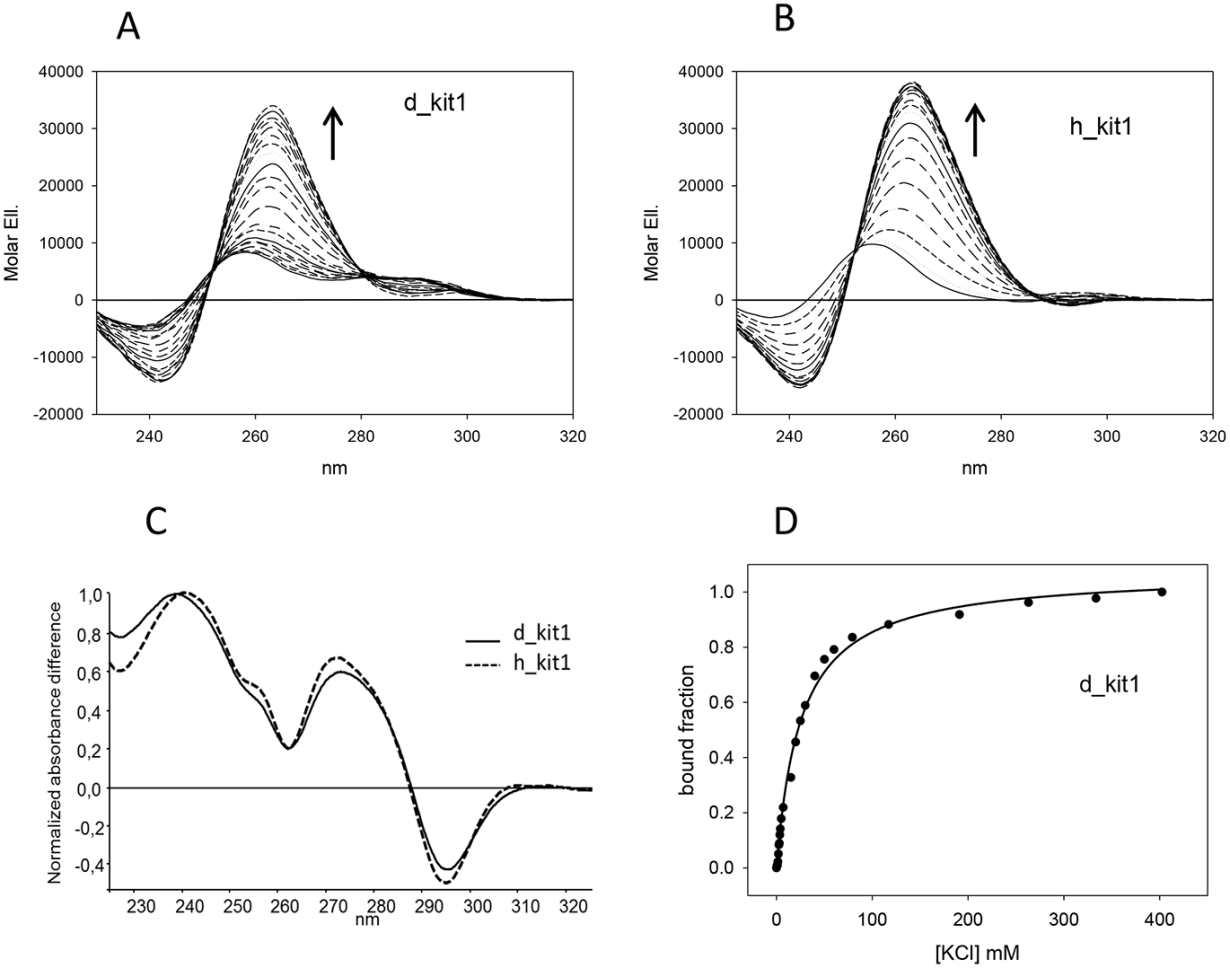
The human and canine kit1 sequences share a common G-4 fold. CD titrations of µM d_kit1 (Panel A) or h_kit1 (Panel B) with increasing concentrations of KCl (0–200 mM) in 10 mM Tris, pH 7.5, 25°C. The induction of G-4 folded form was supported by TDS (Panel C) and quantified from the variation of the CD signal at 260 nm (Panel D).

Monovalent metal ions are known to promote the formation of G-4 structure by G-rich sequences. Among them K^+^ is generally quite efficient and it is also physiologically relevant. Since DNA chiroptical properties are function of its folding topology, we acquired the circular dichroism spectra of the canine sequence d_kit1 upon addition of KCl. The metal ion induced an intense positive band at 260 nm and a negative band at 240 nm (Figure 4A). Once saturated, the CD spectrum of the folded canine sequence was similar to the one obtained after titration of human h_kit1 with KCl (Figure 4B). These features suggest the formation of a parallel G-4 structure which is shared by the two species. This conclusion is further supported by an almost superimposable TDS profile (Figure 4C) [42]. However, the concentration of the metal ion required to saturate the process is remarkably different for the two sequences. A quantitative analysis of the experimental data (Table 3, Figure 4D) confirmed that the canine sequence has a lower affinity for the metal ion. This correlates with the lower thermal stability observed by monitoring the variation of the dichroic signal at 260 nm upon increasing the temperature (Table 4).

**Table 3:** Apparent Kd obtained from CD titrations of the tested sequences with KCl in 10°C.

	Kd (mM)
**d_kit1**	25.62±1.40
**h_kit1**	2.22±0.39
**d_kit2**	1.18±0.11
**d_kit2_A16**	7.58±0.39
**h_kit2**	9.49±0.83

**Table 4:** Melting temperatures of the tested sequences determined from the analysis of the variation of the CD signal recorded at 260°C/min was applied.

	Tm (°C)
**h_kit1**	59.5
**d_kit1**	50.0
**h_kit2**	67.5
**d_kit2**	49.2*
**d_kit2_A16**	43.0*
**h_kit2_T21**	61.4
**h_kit2_T12/21**	63.2
**d_kit2_T12/21**	44.2*
**h_kit2_2tet**	49.0

*values determined at 295 nm.

From these data we can assume that, in potassium containing solutions, the human and canine kit1 sequences share a common parallel G-4 fold, the stability of which is however affected by the second loop composition.

### Canine kit2 exhibits conformational features differing from the human counterpart

Literature data report that, in the presence of KCl, h_kit2 exhibits a highly polymorphic behavior; it folds into at least two main parallel G-4 structures corresponding to a monomeric and a dimeric form. Consequently its dichroic spectrum derives from a combination of these contributions and is characterized by a main positive band centered at 260 nm [37]. This picture is remarkably changed when considering the two canine sequences (d_kit2 and d_kit2_A16).

**Figure 5:**
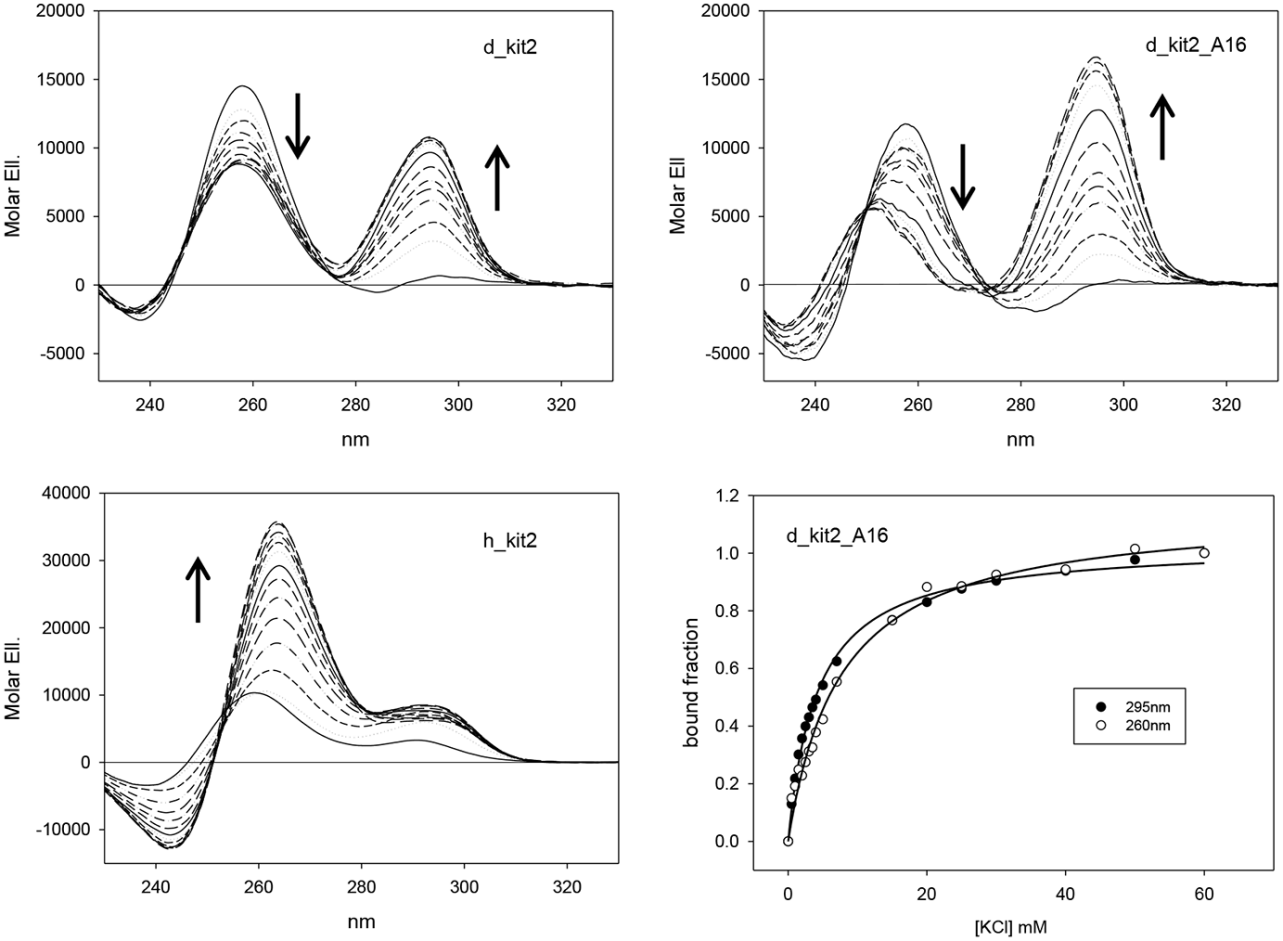
The canine kit2 sequences show distinct chiroptical features. CD titrations of 4 µM canine (d_kit2 and d_kit2_A16 in Panels A and B, respectively) and human (h_kit2 in Panel C) sequences with increasing concentrations of KCl (0–60 mM) in 10 mM Tris pH 7.5, 25°C. In Panel D the relative variations of the d_kit2_A16 CD signal recorded at 260 and 295 as a function of metal ion concentration are reported.

In the absence of the metal ion, a dichroic band centered at 258–260 nm was observed for all tested sequences. However, upon titration with KCl, only the canine ones give rise to a novel contribution at 295 nm associated to a decrease in intensity of the 260 nm signal (Figure 5A–C). The presence of an isodichroic point allows describing the system as composed by two main different optical species which interconvert one into the other in the presence of the metal ion. To confirm this model, we determined the apparent binding constant for the metal ions by monitoring the relative changes of the dichroic signal at 260 nm (Table 3). According to the proposed model, canine data provided very similar figures when analyzed at 295 nm, too (Figure 5D). Thus, the occurrence of a single process affecting the two optical contributions can be inferred. Due to the involvement of KCl we can attribute the 260 and 295 nm bands to the unfolded and G-4 folded fractions, respectively. Hence, the canine sequences apparently fold into a G-4 structure distinct from those assumed by the human sequence. The novel form shows a slightly incremented apparent binding affinity for the metal ion. However, its thermal stability is lower in comparison to the one characterizing the human parallel G-4 in the same experimental conditions (Table 4). The lowest melting temperature was recorded for the mutated sequence d_kit2_A16 in which two guanines expected to be involved in a G-tetrad pairing are mutated into adenines.

**Figure 6:**
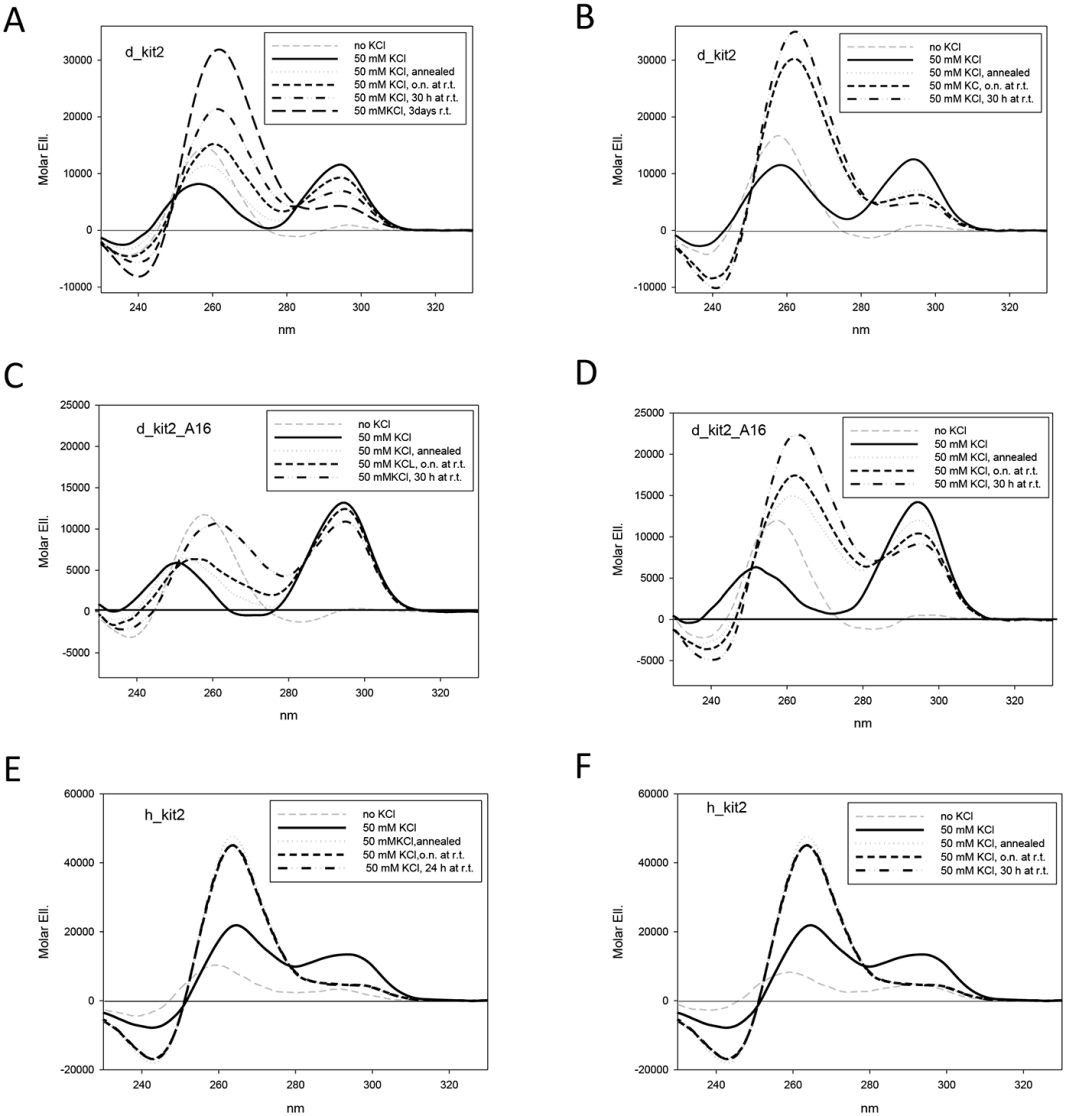
The folding of canine kit2 sequences is affected by sample preparation and DNA concentration. Chiroptical properties of d_kit2, d_kit2_A16 and h_kit2 determined at µM (Panels A, C and E) or 40 µM (Panels B, D and F) strand concentration in 10 mM Tris, pH 7.5, 25°C upon addition of 50 mM KCl.

The relative intensity of the 260 vs 295 nm bands was a function of the DNA sequence. However, we found that sample preparation affected it, too. This is not odd since the simultaneous presence of multiple folded species for the h_kit2 is well established. In particular, both kinetics and DNA strand composition play relevant roles in determining the prevalent folded form of h_kit2. In our experimental conditions, the unfolding transitions of the canine sequences were reversible and no hysteresis was observed using heating rates ranging from 0.4 up to 1°C/min. However, we found that upon incubation in the presence of KCl, a slow rearrangement occurred with decrease of the band centered at 295 nm and concomitant increase of the 260 nm dichroic signal (Figure 6, Panels A and C). The same behavior was observed for the human sequence, for which, however, the process was substantially faster and led to an almost complete suppression of the band at 295 nm in minutes, thus justifying its absence during equilibrium titrations (Figure 6E). At comparable times, the structural conversion is less efficient for the d_kit2_A16 sequence.

### Kit2 intermolecular structures are efficiently formed in the canine sequence

Considering the spectra reported in Figure 6, the slow interconversion of the canine structures seems to fit a two component equilibrium (isodichroic points are present at 278–285 nm, depending upon the tested sequence) which does not involve the unfolded form (the spectra recorded in the absence of the metal ion do not cross at the same isodichroic point). As above introduced, it is well established that the human sequence can rearrange from a monomeric form to a dimeric one. Thus, we decided to assess if for canine sequences the observed rearrangements are connected to the occurrence of a comparable process. To verify this hypothesis, we analyzed the CD features of solutions ranging from 4 up to 40 µM strand concentrations. The results are summarized in Figure 6 where spectra recorded at low DNA strand concentration (4 µM; Figure 6, Panels A, C and E) are compared to those acquired at 10 fold increased concentration (40 µM; Figure 6, Panels B, D, and F).

By increasing the DNA concentration of the two canine sequences, a prevalence of the dichroic contribution at lower wavelength occurs on the equilibrated solutions. As a result, at high concentration the difference in the folding of the sequences deriving from the two species partly vanishes. Again, the metal ion K^+^ is important to promote such an effect since, by reducing its concentration to 20 mM (data not shown), comparable profiles are observed irrespectively of DNA concentration.

**Figure 7:**
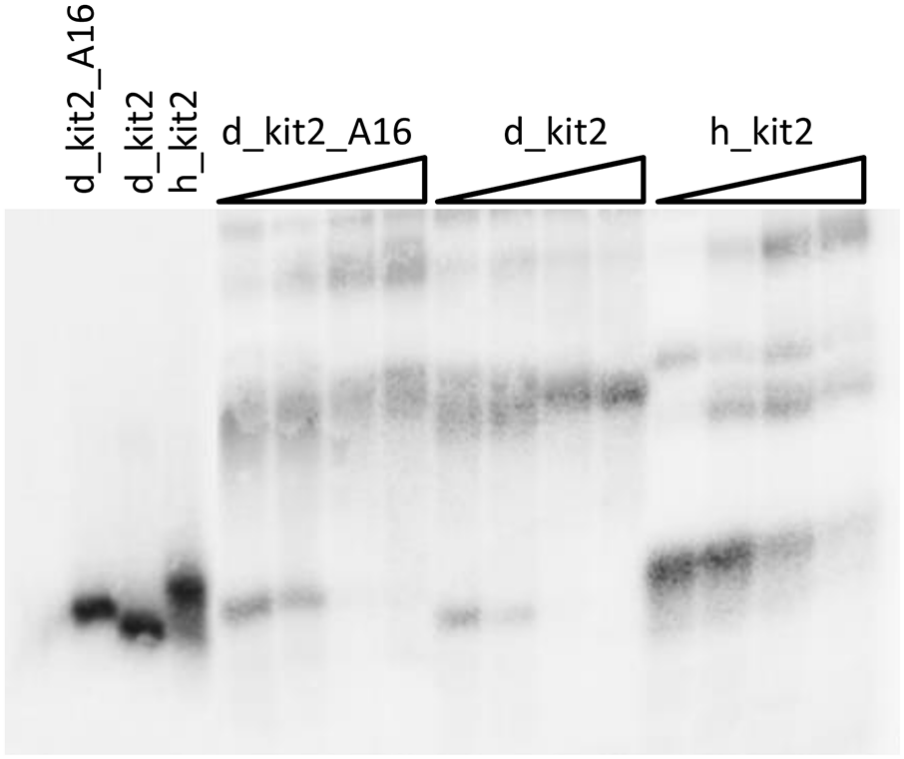
The canine kit2 sequences undergo strand oligomerization. Variation of the electrophoretic mobility of human and canine kit2 sequences upon increasing DNA concentration (0.1–100 µM) in 10 mM Tris, 50 mM KCl, pH 7.5. Lanes 1–3 refer to the purified monomeric forms.

The role of increasing oligonucleotide concentration in modulating the structural features of the tested sequences was confirmed by EMSA. In particular, this assay was applied to resolve the oligonucleotides folded forms according to the number of paired strands, with monomeric forms moving faster (Figure 7). From these gels it emerges that the extent of oligomeric species is well related to the oligonucleotide concentration and is promoted by increasing KCl concentrations (10–100–200 mM). This tendency is more evident with canine kit2 sequences in comparison to the human one.

Interestingly, the resolved products turned out to undergo modest redistribution among forms of different multiplicity once extracted from the gels (Figure S1). Products extraction allowed us to acquire the CD spectra of the purified species (Figure S2). The results confirmed that all multimeric species actually present the CD signature of a parallel G-4 with a main peak located at 260 nm. Nevertheless, none of the resolved forms obtained from the canine or human sequences showed a significant contribution at 295 nm. This rules out the possibility to describe the system with simple monomer-dimer equilibrium. Conversely, it suggests the 295 nm contribution as deriving from a kinetically favored form, which then turns into the thermodynamically stable structure.

### A two-tetrad quadruplex is unlikely for d_kit2 sequences

**Figure 8:**
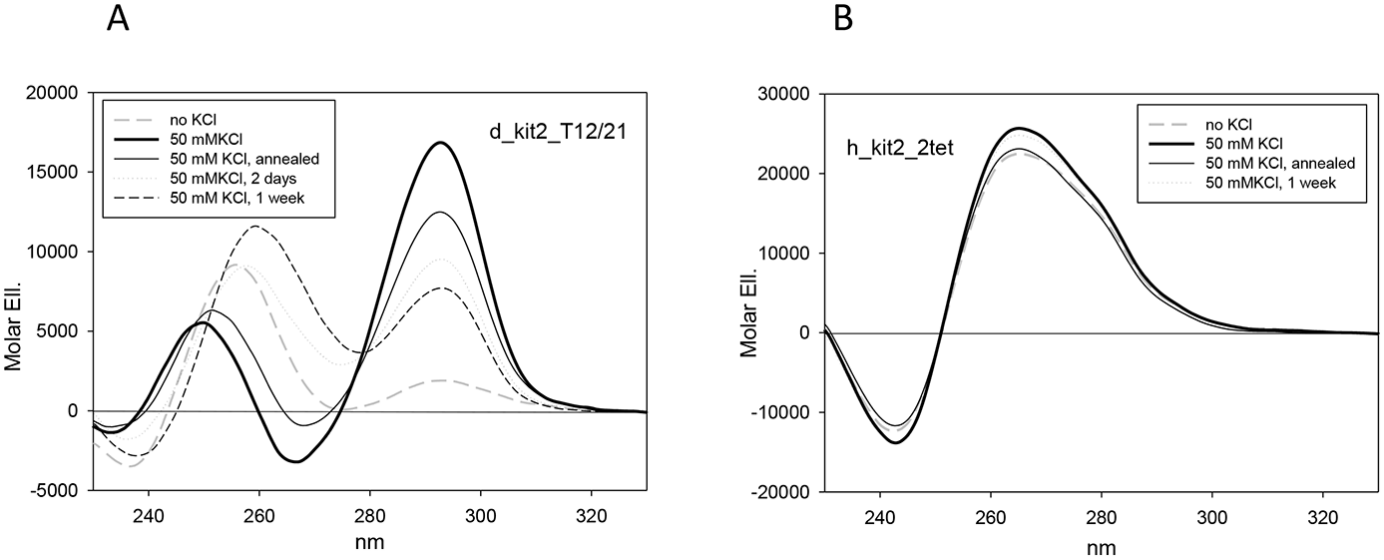
Guanine mutations within kit2 sequences alter folding process. CD spectra of canine and human kit2 mutated sequences recorded in 10°C upon addition of 50 mM KCl.

The heterogeneity of structures assumed by h_kit2 in solution impairs detailed structural studies [37], [43]. However, proper point mutations allowed the prevalent formation of a single structure, thus enabling the acquisition of suitable NMR data. These refer to the sequences h_kit2_T21 and h_kit2_T12/21 (Table 2): they have been confirmed to prevalently fold into a single dimeric or monomeric G-4 structure, respectively, when prepared according to defined experimental conditions (oligonucleotide strand and KCl concentration, time of incubation) [10], [11]. The structure selection by the mutated sequences was obtained by removing guanines which could produce alternative pairings. Thus, we decided to follow a comparable approach on the canine sequence and we analyzed the corresponding d_kit2_T12/21. In analogy to the related wild type sequence d_kit2, in the presence of K^+^, this oligonucleotide showed a CD spectrum composed of two main dichroic bands (260 and 295 nm) (Figure 8A). However, distinctly from the wild type sequence, the conversion of the higher wavelength contribution toward the parallel form(s) is largely impaired. Accordingly, TDSs appear comparable but not perfectly superimposable (Figure S3). Thus we assume that the deleted guanines may be structurally involved in a parallel folded form(s), thus increasing their contribution in the folded population.

**Figure 9:**
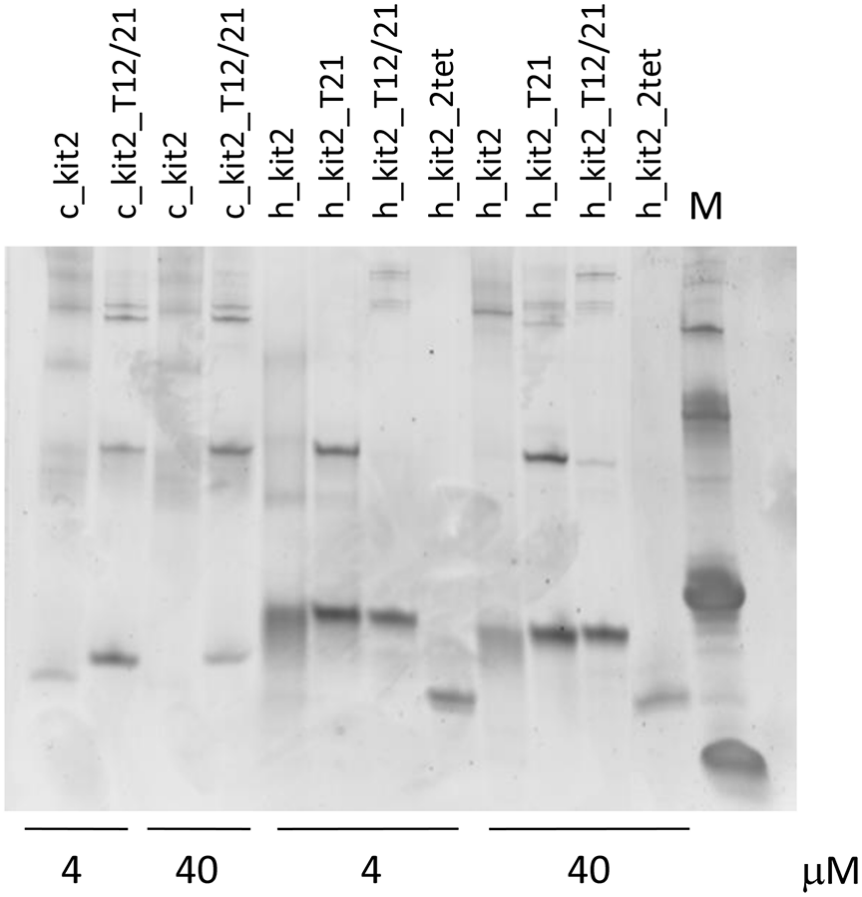
Guanine mutations within kit2 sequences alter electrophoretic mobility of folded forms. Electrophoretic mobility of human and canine kit2 sequences previously annealed at 4 or 40 µM strand concentration in 10 mM Tris, 50 mM KCl, pH 7.5.

Finally, in the canine sequences up to two guanines belonging to one external tetrad of the human parallel G-4 structure (guanines 4, 8, 16 and 20) are lacking. This may be compensated by the recruitment of different guanines along the sequence. Alternatively, the formation of stable G-4 containing just a two tetrads core may be conceived. To assess whether a two-tetrads G-4 may represent a reasonable folding for the canine sequences, we analyzed a mutated sequence derived from the h_kit2_T21 in which all guanines belonging to the external tetrads have been mutated into adenines (h_kit2_2tet). We found that this sequence is actually structurally organized. However, CD and TDS features do not support a G-4 structure but more likely a partly paired hairpin which is not induced by KCl (Figures 8B and S3). Accordingly, it showed a remarkably high migration rate when loaded onto a native PAGE (Figure 9).

## Discussion

In the past decade, the domestic dog has gained increasing interest as the most suitable animal model for comparative oncologic studies on tumor biology as well as for the identification and validation of new therapeutic targets [44]–[48]. Obviously, the robustness of such a model is based, besides the aforementioned histological appearance, tumor genetics, potential prognostic and therapeutic molecular targets and response to conventional therapies, also on the conservation of common cellular pathways and common potentially useful pharmacological targets.

In this connection here we focused our attention on the canine *KIT* promoter region. Overall, to our knowledge this is the first study in which two G-rich portions have been identified at the level of the canine *KIT* promoter region. Additionally, for both of them, the attitude to fold into G-4 structures has been demonstrated. Thus, the analogy with the corresponding human *KIT* gene and the ensuing usefulness as a comparative animal model is confirmed.

As far as kit1 sequence is concerned, the preservation of a common overall parallel G-4 folding between the human and dog derived sequences has been confirmed and is supported by the high degree of sequence homology. The main difference is related to an incremented thermal flexibility of the canine form and to lower efficiency in accommodating potassium ions. This is rationally explained by the disruption of the Watson-Crick base-pair between A1 and T12 occurring in the G-4 folded human sequence [49]. Thus, overall, our evidences confirm that the two systems are suitable for comparative works.

More complex is the description of the behavior of the two canine kit2 sequences in solution.

In dogs, two isoforms not significantly related to the development of a pathological condition were herein identified. Based on the structural information available, the human sequence folds into G-4 structures most of which derive from three overlapping tetrads, where guanines 2–6-14–18, 3–7-15–19 and 4–8-16–20 are paired [10], [11]. When compared to the human sequence, both canine isoforms present mutations that involve guanines belonging to external tetrad (G 4–8-16–20). Nevertheless, along the sequence, additional guanines are present and they can be recruited for G-4 formation. Two of them are peculiar of the canine sequences (G5 and G13) whereas others are conserved in human sequences (G10, G12 and G21). Thus, the multiplicity of folded structures that are formed in solution also by the h_kit2 may be further extended to the dog.

Our experimental data suggest that the different sequences fold into structures of comparable chiroptical features, the main difference being the relative population distribution. In particular, an unprecedented form providing a contribution at 295 nm turned out to be a kinetically preferred one whereas the contribution at 260 nm takes account both of monomeric and intermolecular parallel forms. It is interesting to remind that, in comparison to the wild type human sequence, multimeric G-4 are more extensively represented in the canine counterparts. We initially assumed that this difference arises from a preferential formation of intermolecular G-quadruplex deriving from stacking of only a two-tetrads array which would be beneficially stabilized in the intermolecular arrangement where it could be easier to allow interaction of consecutive G-4. However, the same arrangement in a mutated human sequence (h_kit2_2tet) was found to destabilize any G-4 form to a large extent. Thus, the recruitment of guanines “theoretically” belonging to the loops in order to fulfill the three tetrad array may be considered a realistic possibility. This is straightforward in the d_kit2_A16 where the third guanine strand span up to 4 consecutive guanines which can be recruited in the G-4 core simply leading to a reduction of the length of the second long and flexible loop. Due to the complex structures distribution in solution and due to the fact that the chiroptical properties may be properly described according to the presence of stacking of guanosines of different (positive band at 290 nm) and same (one negative and one positive band at 240 and 260 nm, respectively) glycosidic bond angles, it is not possible at the moment to safely attribute a defined parallel or antiparallel structure to the different components [50]. In the future, the use of specific ligands as structural probe for these sequences might be useful to get further structural insight.

In conclusion, this work validates the canine c-kit promotorial sequence as a potential anticancer target. Clearly, the potential functional role of the observed G-4 motifs needs now to be assessed at the cellular level. Nevertheless, the herein presented evidences support the canine as a comparative model for human disease. While for kit1 the structural superposition is extensive, some differences may be assumed to occur among the kit2 sequences thus indicating them as a potentially species selective target. In fact, recruitment of guanines belonging to the loops in the human sequence to participate in G-4 formation in the canine kit2 could modify the recognition properties of the newly formed loops to a non-negligible extent and create novel opportunities for therapeutic intervention.

## Supporting Information

Figure S1 **Panel A: resolution of d_kit2_A16 folded forms by native gel electrophoresis of samples annealed at different concentration in 10** **mM Tris, 50** **mM KCl, pH 7.5.** The bands labelled 1–3 were extracted from the gel and loaded on the native gel reported in Panel B.
(DOCX)

Figure S2 **CD spectra of monomeric and dimeric forms of canine and human kit2 sequences recovered after purification by native gel electrophoresis.**
(DOCX)

Figure S3 **TDS of selected d_kit2, d_kit2_T12/21 (Panel A) and h_kit2_2tet determined on the previously annealed sequences in 10** **mM Tris, 50** **mM KCl, pH 7.5.**
(DOCX)
