# Supplementary material for: Sequencing and G-Quadruplex Folding of the Canine Proto-Oncogene KIT Promoter Region: Might Dog Be Used as a Model for Human Disease?
Source: PLoS One. 2014 Aug 1;9(8):e103876. doi: 10.1371/journal.pone.0103876 (PMC4118953; doi:10.1371/journal.pone.0103876)
Supplement: Figure S1 — Panel A: resolution of d_kit2_A16 folded forms by native gel electrophoresis of samples annealed at different concentration in 10 mM Tris, 50 mM KCl, pH 7.5. The bands labelled 1–3 were extracted from the gel and loaded on the native gel reported in Panel B. (DOCX) [file pone.0103876.s001.docx]

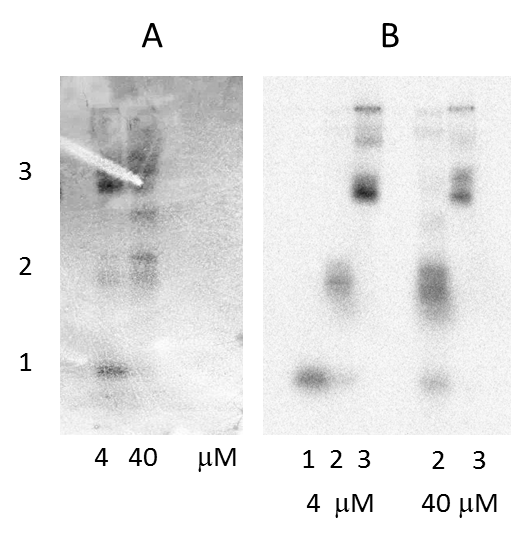


Figure S1. Panel A: resolution of d_kit2_A16 folded forms by native gel electrophoresis of samples annealed at different concentration in 10 mM Tris, 50 mM KCl, pH 7.5. The bands labelled 1-3 were extracted from the gel and loaded on the native gel reported in Panel B.
