# Supplementary material for: Sequencing and G-Quadruplex Folding of the Canine Proto-Oncogene KIT Promoter Region: Might Dog Be Used as a Model for Human Disease?
Source: PLoS One. 2014 Aug 1;9(8):e103876. doi: 10.1371/journal.pone.0103876 (PMC4118953; doi:10.1371/journal.pone.0103876)
Supplement: Figure S2 — CD spectra of monomeric and dimeric forms of canine and human kit2 sequences recovered after purification by native gel electrophoresis. (DOCX) [file pone.0103876.s002.docx]

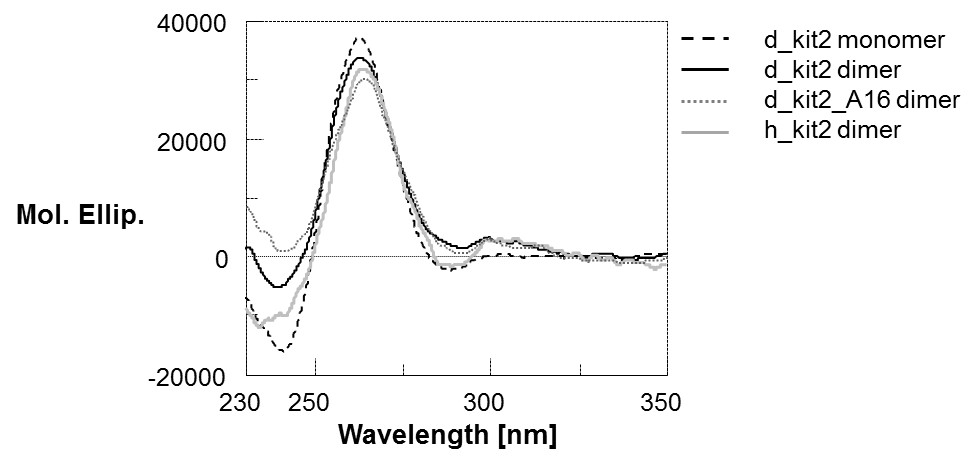


Figure S2. CD spectra of monomeric and dimeric forms of canine and human kit2 sequences recovered after purification by native gel electrophoresis.
