# Supplementary material for: Sequencing and G-Quadruplex Folding of the Canine Proto-Oncogene KIT Promoter Region: Might Dog Be Used as a Model for Human Disease?
Source: PLoS One. 2014 Aug 1;9(8):e103876. doi: 10.1371/journal.pone.0103876 (PMC4118953; doi:10.1371/journal.pone.0103876)
Supplement: Figure S3 — TDS of selected d_kit2, d_kit2_T12/21 (Panel A) and h_kit2_2tet determined on the previously annealed sequences in 10 mM Tris, 50 mM KCl, pH 7.5. (DOCX) [file pone.0103876.s003.docx]

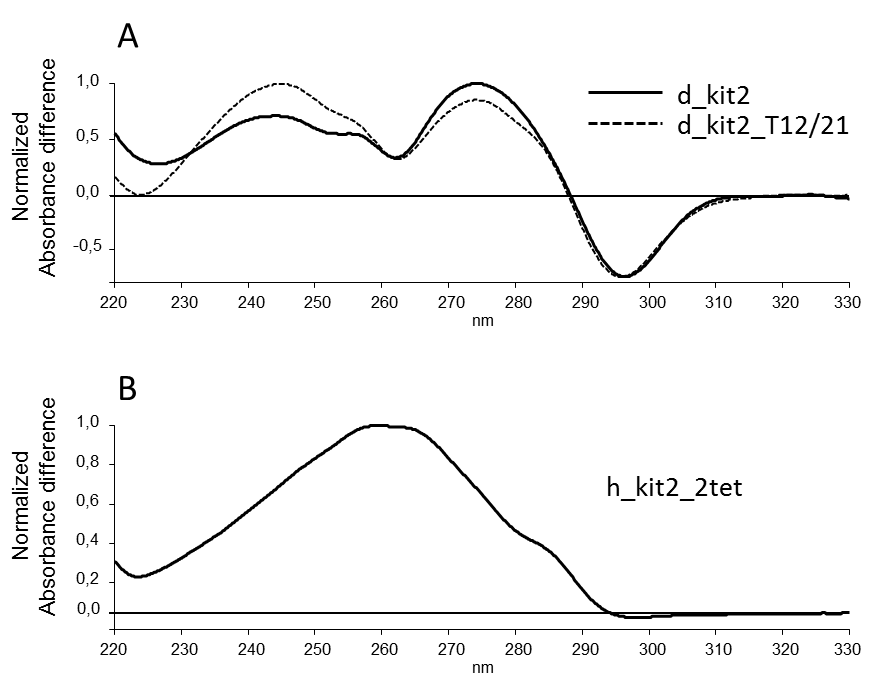


Figure S3. TDS of selected d_kit2, d_kit2_T12/21 (Panel A) and h_kit2_2tet determined on the previously annealed sequences in 10 mM Tris, 50 mM KCl, pH 7.5.
